# The Pancreatic Beta Cell: Editorial

**DOI:** 10.3390/biom13030495

**Published:** 2023-03-08

**Authors:** Alberto Bartolomé

**Affiliations:** Instituto de Investigaciones Biomédicas Alberto Sols, CSIC-UAM, 28029 Madrid, Spain; abartolome@iib.uam.es

Pancreatic beta cells play a critical role in maintaining glucose homeostasis by serving as the primary source of insulin. These cells are responsible for the synthesis, storage, and release of insulin, which is tightly regulated in response to changes in the body’s metabolic status. Due to the central role of beta cells in diabetes pathophysiology, their biology has drawn significant interest from the scientific community. A better understanding of the multiple facets of beta cell biology could lead to developing novel prevention strategies and treatments that can delay or halt disease progression. This Special Issue, “The Pancreatic Beta Cell”, presents a collection of 14 articles, including five original papers and nine reviews, highlighting various aspects of beta cell research. This issue focuses on the molecular mechanisms that control beta cell mass expansion and survival, particularly emphasising significant pathways for mature beta cell function. The contributions cover a broad range of topics, including the impact of oxidative stress on beta cells [1,2,3], inter-tissular communication [4,5,6,7], and master regulators of beta cell mass and function [8,9,10], among others.

Mukai and colleagues [1] reviewed the role of oxidative stress and beta cell antioxidant mechanisms, summarizing how the low expression of antioxidant enzymes in beta cells and oxidative stress can impair insulin secretion. The authors suggested that nuclear factor erythroid 2-related factor 2 (Nrf2) is a master regulator of the beta cell antioxidant response. Wu and colleagues [3] explored the effects of 4-octyl itaconate (4-OI) on pancreatic beta cells under oxidative stress conditions. The researchers found that 4-OI treatment reduced the production of reactive oxygen species, inhibited cell death pathway activation and inflammatory cytokine secretion, and reversed hypoxia-induced cell death, indicating that 4-OI might enhance beta cell survival under oxidative stress conditions.

Additionally, this issue includes articles on the effects of molecules and factors produced in other tissues on beta cell function. Krueger and colleagues [4] studied the effects of the gut microbial metabolite trimethylamine N-oxide (TMAO) on functional β-cell mass. The researchers found that TMAO protected beta cell function and ameliorated oxidative and endoplasmic reticulum stress, despite its reportedly increased levels of type 2 diabetes (T2D) in patients. The authors suggest that TMAO might mediate a compensatory protective effect under diabetogenic conditions. Fernandez-Millan and colleagues [5] discussed the importance of inter-organ communication in the pathology of metabolic diseases such as T2D, emphasizing how understanding the ways beta cells communicate with metabolic and non-metabolic tissues provides a novel area for investigation. They highlight the impact of secreted factors from diverse organs and tissues on beta cell biology, suggesting that inter-tissular communication could afford new opportunities for diabetes research.

The physical proximity of endocrine islets to exocrine cells in the pancreas allows for paracrine interactions between these neighbouring cell types. The impact of exocrine diseases on beta cells is the focus of Ciochina and colleagues’ review [6], which describes how chronic pancreatitis, acute pancreatitis, cystic fibrosis, pancreatic cancer, pancreatic resections, and autoimmune pancreatitis can all affect beta cell function and diabetes progression. The authors emphasize the importance of an early and accurate diagnosis of diabetes in these contexts. Kryvalap and colleagues [7] review the impact of proteases expressed in the exocrine pancreas and serpin protease inhibitors on islet pathophysiology. The authors explore opposing views on the inhibition or augmentation of protease activity in pancreatic islet biology and inflammation and discuss the potential therapeutic targeting of serpins for tissue regeneration and inflammation inhibition in the context of islet dysfunction.

Singh and colleagues [2] reported a potential new therapeutic strategy for enhancing beta cell robustness in type 1 and type 2 diabetes by delivering the anti-apoptotic protein Manf to the beta cells of NOD mice, resulting in a reduced rate of diabetes development. Beta cell hyperplasia during pregnancy is well known, but beta cell effectors are not yet fully understood. Millete and collaborators [11] described the novel in vivo and in vitro models of pseudopregnancy using a hormone cocktail, which could recapitulate the hyperplasic response seen during pregnancy. 

In this issue, several reviews focus on particular signalling pathways or factors that significantly regulate beta cell mass and function. Wang and colleagues [8] summarized the diverse roles of TGF-beta signalling in beta cell development, function, proliferation, apoptosis, and dedifferentiation; conversely, Asahara and colleagues [9] reviewed the impact of beta cell-specific mTOR signalling on beta cell mass and insulin secretion, which could promote the compensatory expansion of beta cell mass and function, but at the expense of the downregulation of homeostatic processes, leading to accelerated cell death. Liang and colleagues [10] review the role of the transcription factor MafA as a significant effector of beta cell functional maturity, focusing on the known transcriptional and post-translational regulators of MafA expression.

Other studies in this issue focus on beta cell function. Ahrén [12] reports a curvilinear relationship between glucose dose and insulin response from murine beta cells in vivo and in vitro, which could be beneficial in improving the design of experiments that aim to explore insulin secretion outcomes. Rohli and colleagues [13] summarize the adaptive compensatory capacities of beta cells that depend on nutritional status, focusing on the cellular processes and molecular regulators that enable adaptive changes and the points of failure leading to T2D progression.

Lastly, Karimova and colleagues [14] provide an insightful review of the current state of protocols to derive islet organoids from human stem cells: a rapidly advancing field in diabetes research with significant therapeutic potential. The authors comprehensively summarized crucial aspects of the molecular pathways involved, the organoid microenvironment, and the current limitations of the protocols, shedding light on the opportunities and challenges facing this emerging technology.

In conclusion, this Special Issue brings together a diverse array of multidisciplinary studies and reviews that underscore the intricate biology of pancreatic beta cells and the complexity of their regulation. The need for continued investigation, collaboration, and a better understanding of the mechanisms underlying beta cell function and dysfunction is highlighted. The insights gained from this research could ultimately pave the way for developing new treatments and prevention strategies for diabetes: a disease in which beta cell dysfunction plays a central role.
